# Anti-glomerular basement membrane glomerulonephritis following nintedanib for idiopathic pulmonary fibrosis: a case report

**DOI:** 10.1186/s13256-017-1384-2

**Published:** 2017-08-06

**Authors:** Ibrahim Ismail, Sonu Nigam, Alan Parnham, Vinay Srinivasa

**Affiliations:** 1 Gold Coast University Hospital, Southport, QLD Australia; 2 Gold Coast University Hospital, Department of Pathology, Griffith University School of Medicine, Southport, QLD Australia

**Keywords:** Anti-GBM disease, Nintedanib, Idiopathic pulmonary fibrosis, Novel targeted therapy

## Abstract

**Background:**

We report a previously unrecognized and unreported case of a patient with anti-glomerular basement membrane glomerulonephritis following nintedanib, an orally active small molecule tyrosine kinase inhibitor.

**Case presentation:**

A 59-year-old Caucasian woman with a history of idiopathic pulmonary fibrosis presented with severe acute kidney injury (creatinine 285 umol/L) secondary to anti-glomerular basement membrane glomerulonephritis disease 4 months after commencement of nintedanib. She had hematuria with red blood cell casts, nephrotic range proteinuria (3.5g/24 hours) and significantly elevated anti-glomerular basement membrane glomerulonephritis titers at 860 chemiluminescent units. A kidney biopsy confirmed severe crescentic glomerulonephritis with linear immunoglobulin G deposition in glomerular basement membrane. Despite the commencement of treatment with plasma exchange and cyclophosphamide, she remained dialysis dependent. Nintedanib was discontinued.

**Conclusions:**

Onset of acute anti-glomerular basement membrane glomerulonephritis was found to be associated with recent nintedanib use suggesting that nintedanib may be a potential trigger for anti-glomerular basement membrane glomerulonephritis. This case highlights the importance of close monitoring of patients receiving new targeted therapies. Management of novel targeted agents in patients receiving dialysis is challenging because of the scarcity of specific data.

## Background

Due to the increasing knowledge of the molecular mechanisms underlying disease and its progression, an ever-increasing number of novel targeted therapies are being developed. The rarer side effects from these relatively newer agents remain unclear and poorly recognized as few dedicated studies are available. While proteinuria and hypertension are well documented adverse events related to targeted therapies, it may be hard to establish a causal link to rarer glomerulonephritides such as anti-glomerular basement membrane glomerulonephritis (GBM) disease. Case reports serve as a means of highlighting the potential association of these rare diseases. To the best of our knowledge, this is the first case report of anti-GBM glomerulonephritis diagnosed following treatment with nintedanib for idiopathic pulmonary fibrosis (IPF).

## Case presentation

**Fig. 1:**
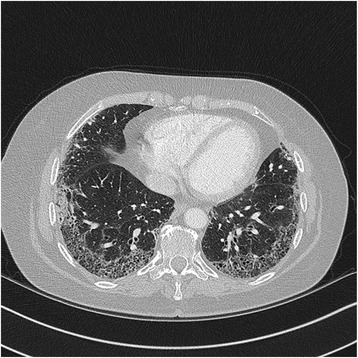
High-resolution computed tomography scan showing extensive peripheral, subpleural pulmonary fibrosis, and early honeycombing

A 59-year-old Caucasian woman was referred to the renal department of our hospital with a 4-week history of painless hematuria and increasing lethargy associated with acute kidney injury (AKI). There was no suggestion of fevers, arthralgia, worsening cough/shortness of breath and in particular, hemoptysis. She had been diagnosed with idiopathic pulmonary fibrosis 4 years earlier, on the basis of radiological and clinical features and was managed by the respiratory department. She had begun treatment with the novel tyrosine kinase inhibitor nintedanib for the last 4 months. High-resolution computed tomography (HRCT) scans showed extensive peripheral, subpleural pulmonary fibrosis, and early honeycombing with superior to inferior gradient in keeping with usual interstitial pneumonia (UIP) (Fig. 1). She also had an extensive investigation for other autoimmune diseases including systemic lupus erythematosis (SLE), sarcoidosis, and rheumatoid arthritis prior to her diagnosis of IPF.

Treatment was complicated by diarrhea that required a dose reduction. Her other comorbidities include a history of breast cancer in remission, Barrett’s esophagus, and osteoporosis. There was no previous history of renal disease. She was an ex-smoker with a 30 pack-year history and consumed less than two standard drinks of alcohol a week.

On examination, our patient was comfortable at rest. Her respiratory rate was 16 breaths per minute, her heart rate was 60 beats per minute, her blood pressure was 90/60 mmHg, saturating at 98% on room air, and she was afebrile. Positive examination findings included dry mucous membranes, clubbing of the fingers, and fine crepitations throughout her chest, consistent with dehydration and pulmonary fibrosis. The jugular venous pressure was not elevated. Heart sounds were dual with no murmurs or pericardial friction rub. Her abdomen was soft and non-tender. There was no pitting edema in the sacrum or peripherally.

### Investigation

**Fig. 2:**
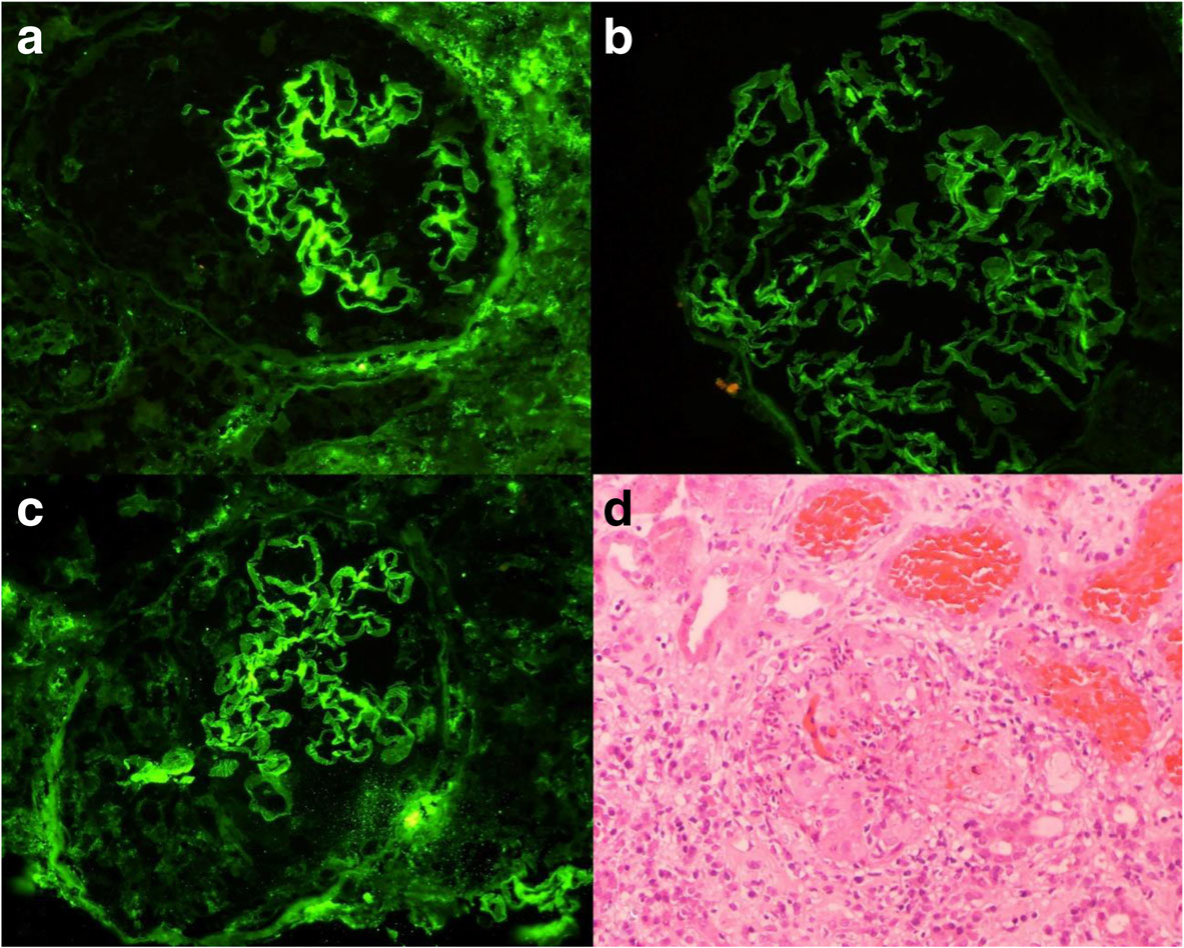
**a**-**c** Linear 3+ reaction along glomerular basement membrane with immunoglobulin G (**a**), kappa (**b**), and lambda (**c**); **d** glomerulus showing cellular crescents associated with inflammatory infiltrate comprised of neutrophils and macrophages, disruption of Bowman’s capsule and fibrinoid necrosis, and red blood cell casts (hematoxylin and eosin ×200)

Her laboratory investigations suggested a nephritic syndrome and AKI. The latter was reflected by a creatinine of 285 umol/L, urea of 8.5 mmol/L, and estimated glomerular filtration rate of 15 mL/min/1.73 m^2^ from a normal baseline renal function 1 month prior. Her urine showed 60 × 10^6^/L leukocytes and greater than 500 × 10^6^/L red blood cells, with evidence of red blood cell casts. Her urinary protein creatinine ratio was 369 g/mol. She had anemia with a hemoglobin level of 91 g/L and hypoalbuminemia with an albumin level of 26 g/L. An enzyme-linked immunosorbent (ELISA) for anti-glomerular basement membrane (anti-GBM) was 860 chemiluminescent units (CU) (<20). Antineutrophil cytoplasm antibodies (ANCA) and antinuclear antibodies were negative and complement levels were normal. A kidney biopsy was promptly performed, which showed evidence of an anti-GBM antibody associated necrotizing crescentic glomerulonephritis with linear deposition of immunoglobulin G (IgG) along the glomerular basement membrane, with 100% crescents (7 out of 7), with rupture of Bowman’s capsule and acute tubular injury (Fig. 2).

### Final diagnosis

The clinical presentation and laboratory findings were consistent with anti-GBM disease.

### Treatment

Our patient was managed as anti-GBM glomerulonephritis initially with pulse intravenous 1 g methyl prednisolone for 3 consecutive days, followed by an oral prednisone 50 mg daily, which was weaned to 20 mg over a month. She was also started on oral cyclophosphamide 150 mg daily and plasma exchange daily (3.5 L exchanges) with fresh frozen plasma for the 5 days’ post-biopsy, followed by plasma exchange with albumin on alternate days for 2 weeks. She required urgent hemodialysis through a temporary dialysis catheter.

### Outcome and follow-up

Initial presenting serum creatinine was 285 umol/L. Our patient’s renal function did not recover and she remained dialysis dependent on discharge from the hospital. Nintedanib was discontinued immediately. Plasma exchange was reduced to weekly from alternate days after the reduction of anti-GBM antibody levels (80 IU) and discontinued at 6 weeks at which point anti-GBM levels were consistently in normal range for the assay (<20 CU). At last follow-up at 12 months, the anti-GBM level has remained low at 13 CU (<20) but our patient remains dialysis dependent receiving thrice weekly in-patient hemodialysis. Cyclophosphamide was ceased at 2 months and prednisolone was gradually weaned to zero over a 6-month period.

## Discussion

Molecular targeted therapy has greatly advanced the treatment of cancers and is being used on a daily basis to treat malignancies. Increasingly, these newer therapies have been used in the treatment of other nonmalignant diseases such as idiopathic pulmonary fibrosis. Nintedanib is a multiple tyrosine kinase inhibitor that works on key angiogenesis pathways including platelet-derived growth factor (PDGF), vascular endothelial growth factor (VEGF), and basic fibroblast growth factor (FGF) [1]. Some of the reported adverse renal effects following the use of other protein kinase inhibitors, especially with older generation agents, include hypertension, proteinuria, and electrolyte disturbances. Endothelial dysfunction is hypothesized as a cause for the development of proteinuria. Renal failure owing to hypertension is a possible adverse reaction of vandetanib, a multikinase inhibitor. The most common histopathological kidney lesion is thrombotic microangiopathy, with other glomerular lesions and interstitial nephritis occurring less frequently [2].

Anti-glomerular basement disease (anti-GBM) was first described by an American pathologist named Ernest Goodpasture in 1919 and is a well-known, albeit rare, cause of glomerulonephritis. Further work from other researchers has led to the identification of the autoantigen responsible for this autoimmune disease [3]. Anti-GBM disease is reported to have the highest rate of crescentic glomeruli at the time of diagnosis [4]. While environmental factors have been suspected to play a role in the pathogenesis others have reported infection as an inciting factor; however, no clear infective pathogenic association has been identified [5, 6]. Multiple case reports also suggest a possible link between hydrocarbon exposures [7, 8]. There are case reports of anti-GBM disease following lithotripsy and ureteric obstruction [9–12]. A number of diseases have been associated with anti-GBM; the most consistently reported association is with membranous nephropathy [13–15]. A previously reported case series demonstrated an association of pulmonary fibrosis and dual myeloperoxidase-antineutrophil cytoplasmic antibody (MPO-ANCA) and anti-GBM positivity [16]. There was no clinical, radiological or biopsy findings for any of these associated causes in our patient: she had no hydrocarbon exposure, did not have a history of recreational drug use or urological procedures, and did not have any preceding infections. She was also negative for a vasculitis screening including ANCA.

Idiopathic pulmonary fibrosis (IPF) is a chronic lung disease with a poor prognosis with a median survival of only 2–3 years after diagnosis [17]. Hence, it has been the subject of substantial research to explore therapeutic targets to decrease the symptoms burden and improve overall survival. Nintedanib was approved by the United States Food and Drug Administration (FDA) for the treatment of idiopathic pulmonary fibrosis in October 2014 following successful phase III clinical trials [18].

There are a number of reports of VEGF inhibitor-induced kidney damage including crescentic glomerulonephritis [2]. There is a growing body of evidence that suggests multikinase inhibitors not only heavily impact the immune system, and further new evidence has emerged implicating its role in vascular endothelium dysfunction and glomerular epithelial cell (podocyte) dysregulation [2, 19]. Studies done in animal models have shown the suppression of VEGF activity resulting in the interruption of glomerular development leading to abnormal glomeruli, which are deficient in capillaries but with an increase in the matrix [19]. In abnormal glomeruli, no endothelial cells are seen by electron microscopy [19]. It is possible the change in the glomeruli architecture is enough to expose GBM antigens and consequently, antibody formation. Studies done in mice models with anti-GBM glomerulonephritis, demonstrating low tissue VEGFR, VEFGR-2, Ang-1 and Tie 2 expression and treatment with VEGF_165_ led to improvement of renal function and proteinuria though recovery of crescentic lesions, proliferation of endothelial cells, and capillary repair. Data from human studies have also demonstrated the importance of VEGF in glomerular recovery in crescentic glomerulonephritis by stimulating capillary endothelial cell growth and proliferation [20].

Interestingly, pirfenidone, an antifibrotic agent approved for use in idiopathic pulmonary fibrosis, has been explored as a renoprotective agent in diabetic nephropathy and focal glomerular disease [21, 22]. There is an overlap in adverse event profiles of pirfenidone and nintedanib, however, no specific cases of glomerulonephritis associated with either drug in the literature. Unlike pirfenidone, whose mechanism of action is not fully elucidated, nintedanib is a potent multikinase inhibitor that blocks VEGF receptors among others. Furthermore, experimental animal models suggest both platelet-derived growth factor (PDGF) and fibroblast growth factor (FGF) are involved in the evolution of crescentic glomerulonephritis and scar formation. The antagonism of these factors (PDGF and FGF) by giving nintedanib would theoretically have a beneficial effect on the damaged glomeruli [23].

Despite nintedanib being increasingly used to treat patients with IPF, this is the first reported case of an association with anti-GBM. This could be because anti-GBM disease is not only a rare disease but also the anti-GBM antibodies are intrinsically heterogeneous with respect to collagen type IV reactivity [24–26]. Furthermore, patients with chronic kidney disease are almost always excluded from large clinical trials [27]. Given these patients have co-existing chronic comorbidities, it is vital to focus on safety and efficacy of these newer targeted therapies. While nintedanib may have triggered anti-GBM in our patient, we have no direct proof of causality. Nintedanib was discontinued in our patient not only because we hypothesize it triggered her anti-GBM but also because there is no data for its use in a patient on dialysis.

## Conclusions

As more novel agents are identified and increasingly used renal toxicities will become more prevalent and robust pharmacovigilance programs are essential to identify the rarer adverse events of these drugs.

## Abbreviations

AKI: Acute kidney injury
ANCA: Antineutrophilic cytoplasmic autoantibodies
Anti-GBM: Anti-glomerular basement membrane
CU: Chemiluminescent unit
ELISA: Enzyme-linked immunosorbent essay
FDA: Food and Drug Administration
FGF: Fibroblast growth factor
H&E: hematoxylin and eosin stain
HRCT: High-resolution computed tomography
IgG: Immunoglobulin G
IPF: Idiopathic pulmonary fibrosis
PDGF: Platelet-derived growth factor
RBC: Red blood cell
SLE: Systemic lupus erythematosus
UIP: Usual interstitial pneumonia VEGF, Vascular endothelial growth factor
